# Neuropsychological Assessment for Early Detection and Diagnosis of Dementia: Current Knowledge and New Insights

**DOI:** 10.3390/jcm13123442

**Published:** 2024-06-12

**Authors:** Patricia Alzola, Cristóbal Carnero, Félix Bermejo-Pareja, Gonzalo Sánchez-Benavides, Jordi Peña-Casanova, Verónica Puertas-Martín, Bernardino Fernández-Calvo, Israel Contador

**Affiliations:** 1 Department of Basic Psychology, Psychobiology and Methodology of Behavioral Sciences, University of Salamanca, 37005 Salamanca, Spain; patricialzola@usal.es; 2 Neurology Department, Granada University Hospital Complex, 18014 Granada, Spain; 3 Centro de Investigación Biomédica en Red de Enfermedades Neurodegenerativas (CIBERNED), Institute of Health Carlos III, 28029 Madrid, Spain; 4 Institute of Research i+12, University Hospital “12 de Octubre”, 28041 Madrid, Spain; 5 Barcelonabeta Brain Research Center, 08005 Barcelona, Spain; 6 ‘Hospital del Mar’ Medical Research Institute, 08003 Barcelona, Spain; 7 Faculty of Education, International University of La Rioja, 26006 La Rioja, Spain; 8 Department of Psychology, University of Córdoba, 14071 Córdoba, Spain

**Keywords:** dementia, Alzheimer’s disease, neuropsychological assessment, early detection, cognitive decline, differential diagnosis

## Abstract

Dementia remains an underdiagnosed syndrome, and there is a need to improve the early detection of cognitive decline. This narrative review examines the role of neuropsychological assessment in the characterization of cognitive changes associated with dementia syndrome at different states. The first section describes the early indicators of cognitive decline and the major barriers to their identification. Further, the optimal cognitive screening conditions and the most widely accepted tests are described. The second section analyzes the main differences in cognitive performance between Alzheimer’s disease and other subtypes of dementia. Finally, the current challenges of neuropsychological assessment in aging/dementia and future approaches are discussed. Essentially, we find that current research is beginning to uncover early cognitive changes that precede dementia, while continuing to improve and refine the differential diagnosis of neurodegenerative disorders that cause dementia. However, neuropsychology faces several barriers, including the cultural diversity of the populations, a limited implementation in public health systems, and the adaptation to technological advances. Nowadays, neuropsychological assessment plays a fundamental role in characterizing cognitive decline in the different stages of dementia, but more efforts are needed to develop harmonized procedures that facilitate its use in different clinical contexts and research protocols.

## 1. Introduction

The term dementia, which has a Latin origin (“without mind”), has undergone changing definitions throughout history [1]. Today, in a nutshell, it is defined as a syndrome consisting of signs and symptoms caused by a variety of diseases that eventually lead to significant cognitive, behavioral, and functional impairments [2,3]. The fifth edition of the Diagnostic and Statistical Manual of Mental Disorders (DSM-5) provides an updated framework for diagnosing dementia under the name of “Major Neurocognitive Disorder”, which offers a broader medical definition than the classical dementia concept [4]. Essentially, there must be a significant cognitive decline from a previous level of performance in one or more cognitive domains (i.e., memory impairment is no longer essential for diagnosis), with a significant interference in activities of daily living (ADLs). This evidence should be based on the concern of the individual or a reliable informant (e.g., relative, clinician) and objectifiable by standardized neuropsychological testing. In addition, it is necessary to specify whether the disorder is accompanied by a behavioral disturbance [5]. It is noteworthy that the number of people living with dementia worldwide is expected to increase from 57.4 million in 2019 to nearly 153 million in 2050 [6]. Therefore, there is a critical need for public health planning efforts and policies to address the needs of this population.

Dementia-related brain changes are known to begin years before the clinical diagnosis [7]. Thus, several entities have been described that attempt to define the intermediate (i.e., predementia) state between normal cognitive aging and dementia [8], as shown in Table 1. Among these predementia constructs, “mild cognitive impairment” (MCI) is the one that has gained greater popularity [9]. The term MCI was proposed by Petersen et al. [10] to identify subjects with isolated memory problems who showed an increased probability of developing Alzheimer’s disease (AD). The International Working Group on Mild Cognitive Impairment (IWGMCI) updated this construct, pointing out that subjective complaints are no longer required and that other cognitive domains (e.g., language, executive functions) may be affected independently of memory, resulting in different cognitive phenotypes [11,12]. These new criteria include the distinction between amnestic and non-amnestic MCI subtypes and whether cognitive impairment is limited to a single domain or multiple domains, providing etiological and prognostic characterizations of clinical utility. The IWGMCI consensus provided a flexible framework for MCI diagnosis and agreed that biomarkers could help elucidate clinical progression [12]. Given that access to biomarkers is usually very limited in diverse community settings (e.g., low-income countries) [13], neuropsychological assessment may play an important role in identifying cognitive risk profiles in the prodromal stages of neurodegenerative diseases and dementia diagnosis [14,15,16]. 

Currently, subjective cognitive decline (SCD), described as subjective perceptions or complaints of cognitive impairment not detected by neuropsychological testing, is an increasingly common medical concern [20,21]. In this context, ~40% of people aged 65 years or older experience some form of memory loss [21]. Even when SCD does not exceed the cutoffs of neuropsychological tests for objective cognitive impairment, this condition has been associated with an increased risk of cognitive decline and dementia [22,23]. The identification of this condition, as well as the detection of subtle cognitive changes, remains a challenge in healthcare systems [24]. Some of the reasons include time constraints, lack of collaboration among healthcare professionals, physicians’ difficulty differentiating between normal aging and MCI, or lack of expertise in cognitive assessment [24,25,26,27]. In addition, caution must be taken when assessing the cognitive status of minority samples (e.g., poorly educated, non-Western patients), as cultural factors such as schooling, administration language, or culturally influenced items may impact cognitive performance, leading to higher rates of false positives for dementia [28,29,30].

The aim of this narrative review was to analyze and synthesize current knowledge in the field of the neuropsychological assessment of aging and dementia. First, we examined the key cognitive markers of cognitive decline and the main barriers to early detection. We also addressed the use of screening instruments in clinical care settings. Second, we proposed a brief guideline to streamline the differential diagnosis of AD and other dementias. Finally, we discussed the current challenges of neuropsychological assessment in aging and dementia providing new insights for future research.

## 2. Methods

The present study is a narrative review of a complex and extensive topic. Articles were selected from sequential PubMed searches using the criteria for “best match” and most recent articles. The MeSH (Medical Subject Headings) were “dementia and early diagnosis”, which produced ~15,000 references. Also, we explored other MeSH such as “subjective cognitive decline and cognitive tests”, and “predementia and cognitive tests”. We also consulted the *Cochrane Book for Systematic Reviews and Meta-Analysis Protocols* checklist for guidance on the standard methods for reviews [31], and the QUADAS recommendations for evaluating test diagnostic accuracy [8,32]. Further, we reviewed the main expert consensus, task forces, and recommendations about these subjects, either in journals or in books. Cited or related articles were also considered for the study. We included mostly English literature, but Spanish papers were also studied, including journals of different Spanish societies such as neurology, psychiatry, psychology, and geriatrics in the last ten years. The authors (neurologists and psychologists) are experts in the field who work at different hospitals and universities in Spain. They discussed the bibliography obtained and agreed on the final text. This review is in accordance with the Scale for the Assessment of Narrative Review Articles (SANRA) [33].

## 3. Detection and Screening for Cognitive Impairment

### 3.1. Early Detection of Cognitive Changes

Cognitive decline is known to be associated with the aging process, but individuals may experience it at different rates [34,35]. Given this interindividual variability, predementia constructs (i.e., intermediate states between healthy cognitive aging and dementia) have been a research topic of great interest. This fact has led to the window of preventing dementia, especially AD, from its earliest stages [8]. Accordingly, many pharmacologic and non-pharmacologic therapies have been used in individuals with predementia (e.g., MCI) to achieve this goal [36,37,38]. Two recent meta-analyses estimate that MCI affects more than 15% of people over 50 [39,40]. Notably, progression rates from MCI to dementia vary widely, from <1% to >50% [41]. These results may depend on the sociodemographic characteristics of the population (e.g., age, sex, and income) and the study setting (e.g., clinical vs. community), among others. Although individuals with MCI have an increased risk of developing dementia [42,43], some of them remain stable over time (~37% to 67%) [44], or even return to cognitive normality (~8% to 25%) [45,46]. 

The diagnosis of predementia or prodromal stages requires extensive biological, neuroimaging, and clinical assessment, including a comprehensive neuropsychological examination, which plays a prominent role [47]. The differentiation of predementia stages from cognitively normal individuals or mild dementia cases remains a challenge, especially in the general population [48,49,50]. Although comprehensive evidence-based guidelines for preclinical cognitive assessment do not yet exist, research on the prodromal dementia stages (particularly AD) has begun to delineate its earliest cognitive manifestations [51,52,53]. In this context, the use of cognitive tests is proving useful in distinguishing cognitively healthy individuals from those in the stages of SCD, predementia, and mild dementia [54,55]. For instance, novel neuropsychological test paradigms (e.g., face–name associative memory, spatial pattern separation) show good accuracy in discriminating between healthy controls and participants with SCD [54]. 

In recent years, research has demonstrated that physiological and cognitive changes are indeed present in pre-MCI individuals with AD biomarkers [56]. In this context, significant changes in tasks assessing a variety of cognitive functions (e.g., episodic memory, semantic memory, language, and perception) have been found in the preclinical stage of AD [57]. Likewise, low cognitive performance on memory, attention, and executive tasks (i.e., Paragraph Initial Recall, Digit Symbol Test, and WAIS Digits Forward) predicts future decline, even in individuals at GDS stage 1 (i.e., without subjective or objective decline) [58]. 

In people with SCD, performance on long-term verbal memory tasks have been suggested as reliable indicators for estimating the risk of progression to AD [59]. In addition, global cognitive function and psychomotor speed have also been proposed as predictors of AD-type dementia in individuals with SCD and MCI [60]. In fact, verbal memory measures in combination with other cognitive domains (e.g., language, executive function, visual memory) have been associated with amyloid deposition and hypoconnectivity across brain networks, becoming excellent indicators of progression from MCI to AD [52,61]. Specific cognitive patterns (e.g., asymmetry in cognitive test performance) and the progression of neuropsychological changes may also serve as important markers of early disease, even before significant cognitive impairment occurs [62]. Taken together, these findings suggest that the study of cognitive profiles may assist clinicians in predicting an individual’s cognitive progression to dementia [59,63,64,65].

It is worth noting that a comprehensive neuropsychological assessment, rather than a single screening test, is critical for distinguishing participants with SCD from those without self-reported cognitive complaints, or MCI from mild dementia [22,66,67]. Neuropsychological assessment may provide clinicians promising markers of neurodegenerative diseases that cause dementia in a less invasive and cost-effective manner [68,69]. Nevertheless, early cognitive markers require further investigation to promote their appropriate implementation in clinical settings [70]. 

An early diagnosis of dementia would potentially allow the involvement and support of the patient’s family, the initiation of treatments, and the delay of the patient’s institutionalization, ultimately reducing the healthcare costs associated with home and specialized services [71,72]. However, some perspectives suggest that the early diagnosis of dementia (e.g., at preclinical stages) in the absence of an effective treatment is not sufficiently justified, increasing the suffering of the patient and family due to stigma and the anticipation of progressive disability [73,74]. In this context, some authors argue that a “timely diagnosis” (i.e., at the time of symptom onset) may be a more appropriate option [75]. Nevertheless, the public health system faces different challenges, including the lack of training in primary care services, delays of referrals, and difficulties in the transdisciplinary management of cases, which hinder the timely diagnosis of dementia [76,77,78,79]. These facts suggest the need to promote public and professional education on dementia diagnosis and improve the collaboration between health services.

### 3.2. Screening for Cognitive Impairment

Cognitive impairment (CI) refers to a significant decline in cognitive performance from a previous level, which is greater than expected by age. This decline is independent of age onset (e.g., CI in young adults), etiology (e.g., AD vs. stroke), clinical expression (i.e., cognitive profile), and degree of functional impairment (e.g., little or none in MCI, or significant in dementia). Further, a CI diagnosis should not depend on education and cultural factors (i.e., language of administration) or the interaction between the clinician and the examinee [29,80]. 

Despite the high prevalence, CI is underdiagnosed and affected individuals are largely unrecognized [81]. Currently, there is no evidence to justify population screening in asymptomatic individuals [82,83,84,85,86]. However, this is not incompatible with encouraging clinicians to be alert and sensitive to early signs of CI. In this context, a brief and accurate cognitive assessment is recommended to ensure early detection, which has shown benefits for the patients, family, and society [87,88]. To achieve this goal, cognitive instruments should demonstrate sufficient scientific rigor, including robust sensitivity, specificity, and predictive utility in culturally and linguistically diverse populations [51].

Given the time constraints in clinical practice, cognitive assessments are usually carried out through the application of Brief Cognitive Tests (BCTs). Basically, BCTs should have the following characteristics and conditions [89]. First, screening tests should be brief considering the setting and circumstances. For instance, in some general neurology consultations, instruments that take more than 15 min could be used, whereas in primary care, only instruments that take less than 5 min are suitable. Second, BCTs should be simple, accessible, and easy to use, requiring little instrumentation (i.e., paper and pencil) and training for its application. For this reason, it is also highly recommended that screening tests be freely available and free of charge. Third, in terms of population, screening tests should be applicable to all individuals, including illiterate/poorly educated individuals and those from other cultures. Thus, it will be necessary to have adequate normative values (e.g., age- and education-adjusted). In addition, BCTs should be adapted and validated in the context that they will be used. In fact, the Mini-Mental State Examination (MMSE; [90]) has shown differences in sensitivity and specificity depending on the application setting [91]. This author showed that MMSE is reasonably effective in identifying dementia in specialized settings (e.g., memory clinics), but should not be used alone if the screening for dementia was negative. Conversely, in non-specialized settings (e.g., primary care), the only value of the MMSE is to rule out dementia in individuals with memory concerns. Finally, efforts should be made to include a multidomain examination (vs. exclusive memory-oriented evaluation), which could help to elucidate different and subtle forms of CI. 

Table 2 depicts a list of widely known international screening tests, indicating whether they meet the above characteristics. In particular, it specifies the approximate time of administration, the domains assessed, whether it is freely available, the validation context (clinical or population-based setting), and the extent to which it is used in different countries. The reader should consider that there have been modifications of the original versions of each test over time, which are not mentioned. A paradigmatic example could be the 30-point MMSE [90], but there are also short (MMSE-12 and MMSE-20) [92], standardized [93], and longer versions in different languages [94,95], including the 100-point MMSE [96]. Moreover, we encourage clinicians to also include functional tests in the screening practice, especially in the presence of a reliable informant. The Functional Assessment Questionnaire [97], 8-item informant interview (AD8; [98]), or the Lawton IADL [99] are some examples. The table is intended to serve as a guide for the cognitive screening of people under suspicion of CI in various healthcare settings (e.g., primary and specialized medical settings). In this regard, clinicians should critically evaluate the appropriateness of these tests for their application context and the characteristics of the patients that will be assessed [100]. 

In addition to the BCTs listed in Table 2, other tests widely used in in Spain include the Memory Alteration Test (M@T) [125], the Eurotest [126], and the Leganes Cognitive Test (PCL) [127]. These tests have demonstrated adequate sensitivity and specificity in detecting MCI and dementia (see [128,129,130,131] for a review).

## 4. Neuropsychological Assessment and Differential Diagnosis of Dementia

Detecting the onset of cognitive changes and distinguishing between different etiologically related disorders can be challenging. As the most common form of dementia, AD has been widely studied in the neuropsychological research field. However, this neurodegenerative disease is not the only proteinopathy that destroys healthy neurons and their connections [132,133]. Understanding the discrete cognitive, behavioral, and emotional profiles that arise from each disorder can help clinicians to distinguish among various types of dementia and suggest more precise rehabilitation strategies and programs. The following sections describe different forms of dementia and how they can be cognitively differentiated from AD. A general overview of common cognitive performance by type of dementia and cognitive domain is provided in Table 3. This knowledge should always be adapted to each patient and weighed against clinical judgment and the gold standard diagnosis for each neurodegenerative disease.

### 4.1. Alzheimer’s Disease

AD is an age-related degenerative brain disorder characterized by the abnormal accumulation of amyloidogenic plaques and neurofibrillary tangles in the brain, causing synapse loss and neuronal atrophy [173,174]. AD is the most common form of dementia, accounting for 60% to 80% of all dementia cases [25]. The most commonly used diagnostic criteria to characterize this disease have been established by the joint working group of the National Institute of Neurological and Communicative Disorders and Stroke (NINCDS) and the Alzheimer’s Disease and Related Disorders Association (ADRDA) [175], with recent updates provided by the National Institute on Aging—Alzheimer’s Association (NIA-AA) [174]. According to these working groups, the core clinical criteria of dementia due to AD includes (a) insidious onset; (b) clear-cut history of worsening cognition; (c) prominent cognitive deficit in memory (amnestic presentation), language, visuospatial skills, or executive functions (non-amnestic presentations); and (d) the diagnosis should not be applied in the presence of other substantial concomitant diseases (e.g., cerebrovascular disease). Cognitive dysfunction is usually accompanied by psychological and behavioral disturbances (e.g., personality changes, anxiety, depression, emotional lability, agitation, delusions), leading to a marked decline in daily functioning [171]. 

Typically, AD is characterized primarily by episodic memory impairment, temporal and spatial disorientation, and language and executive dysfunction, leading to subsequent impaired social functioning [154]. However, several studies report that AD is not a homogeneous entity, describing four atypical or non-amnestic forms (i.e., logopenic variant primary progressive aphasia (lvPPA), posterior cortical atrophy (PCA), behavioral/dysexecutive variant (bvAD), and corticobasal syndrome (CBS). These forms differ from typical AD in age of onset and clinical presentation and may be related to underlying biological subtypes [176,177]. For this reason, a comprehensive clinical history and neuropsychological assessment focusing on orientation, attention, language, praxis, memory, visuospatial skills, gnosis, and executive function are critical to characterize the patient’s cognitive presentation and differentiate it from the typical profiles of various diseases [178]. 

### 4.2. Parkinson’s Disease

Parkinson’s disease (PD) is characterized by the progressive loss of dopaminergic neurons of the substantia nigra pars compacta (SNpc) of the midbrain, as well as the presence of intracellular inclusions (i.e., Lewy bodies) [179]. This neurodegeneration manifests as slowness in initiating voluntary movements with progressive slowing (bradykinesia), accompanied by muscle rigidity, resting tremor, and/or postural instability unexplained by other causes (e.g., visual or vestibular dysfunction) [180]. Besides the classic motor symptoms, non-motor features like cognitive dysfunction are now widely accepted as part of the clinical profile of the disease [181]. The prevalence of dementia in PD (PD-D) is estimated to be between 20% and 40% [182,183]. Currently, the Movement Disorder Society is a working group that provides clinical diagnostic criteria for probable and possible PD-D [184]. They describe the core features as (a) a diagnosis of PD according to Queen Square Brain Bank criteria, and (b) an insidious and slowly progressive dementia syndrome (i.e., impairment in more than one cognitive domain, decline from premorbid level, and compromised ADL), which develops in the context of established PD and is diagnosed by history, clinical, and mental examination. This profile can be associated with other behavioral features like apathy, changes in personality and mood (e.g., depression, anxiety, or emotional incontinence), hallucinations (mostly visual), delusions and excessive daytime sleepiness [143].

The expected cognitive features of patients with PD-D are fluctuating attention, deficits in executive functions, visuospatial abilities, and verbal and visual memory, with mostly preserved language function [136,183]. Hence, tests related to executive functions (i.e., mental flexibility, planning, conceptualization, and abstraction abilities), as well as attention and visuospatial tasks, are recommended [161]. Moreover, memory and language tests may be essential to differentiate the PD cognitive profile from cortical dementias such as AD. In this sense, patients with AD exhibit deficits in cognitive domains such as episodic memory, praxis, language, and arithmetic, whereas patients with PD-D tend to exhibit slower mental processing speed, dysexecutive deficits, and visuospatial and visuoconstructional deterioration, accompanied by frontal lobe alterations such as apathy and irritability [143,145,157,185]. The use of neuropsychological scales, such as the Scales for Outcomes in Parkinson’s disease-Cognition (SCOPA-Cog) [186] and the Parkinson’s Disease-Cognitive Rating Scale (PD-CRS) [187], could help clinicians identify patterns of cognitive impairment associated with PD. 

### 4.3. Dementia with Lewy Bodies

Dementia with Lewy bodies (DLB) is a disease associated with abnormal deposits of alpha-synuclein protein in the brain (i.e., Lewy bodies). These deposits affect brain chemicals, which can cause fluctuating cognitive impairment, visual hallucinations, extrapyramidal motor features, and sleep behavior disorder [188]. Approximately 5% of older people with dementia present evidence of DLB only, although most people with DLB also have AD pathology [25]. The diagnosis of DLB is based on the revised consensus reached by the Consortium on Dementia with Lewy Bodies [144]. This working group stablish the following core clinical features: (a) fluctuating cognition with pronounced variations in attention and alertness; (b) recurrent and detailed visual hallucinations; (c) sleep disorder; (d) one or more spontaneous cardinal features of parkinsonism (i.e., bradykinesia, rest tremor, or rigidity). These symptoms occur early and may persist during the course of the disease. 

DLB has overlapping clinical and neuropathologic features with AD and PD-D, making the differential diagnosis complex. Concerning PD-D, a distinction has been proposed regarding the timing of the onset of motor and cognitive symptoms, as DLB is associated with cognitive impairment from the early stages and extrapyramidal motor features are often mild or absent until the late stages, whereas in PD-D, early and prominent extrapyramidal motor features are present first, with neuropsychiatric and cognitive symptoms appearing later [188]. Nonetheless, the distinction between PD and DLB is still a matter of controversy [189]. Meanwhile, AD is distinguished from both conditions by the fact that DBL and PD-D may show marked fluctuations in cognitive impairment and by the high prevalence of visual hallucinations [136]. Moreover, patients with DLB often have disproportionately severe deficits in executive function (i.e., initiation, perseveration, mental flexibility, and working memory), attention (i.e., selective, divided and sustained), and perceptual motor processing, whereas their memory (i.e., verbal retention and recognition) and language (i.e., naming) deficits are generally less severe than in patients with AD [145,146]. The combination of attentional and visuoperceptual dysfunction, combined with relative preservation of memory and naming, can help differentiate DLB from AD in the early stages with substantial sensitivity (83.3%) and specificity (91.4%) [190]. In addition, the use of the Clinician Assessment of Fluctuation (CAF) [191] may help to capture fluctuating cognition and play a valuable role in differentiating DLB from AD [192]. 

### 4.4. Vascular Dementia

Vascular dementia (VaD) is understood as a cumulative decline in cognitive functioning secondary to multiple or strategically placed infarctions [193]. It is the most severe form within the spectrum of vascular cognitive impairment (VCI), defined as any cognitive condition caused by or associated with vascular factors [194,195]. According to estimates, 5 to 10% of individuals with dementia show evidence of vascular dementia alone, with mixed dementias being the most frequently observed [196]. One of the most widely used diagnostic criteria for VaD has been the National Institute of Neurological Disorders and Stroke–Association Internationale pour la Reserche et l’Enseignement en Neuroscience (NINDS/AIREN) [197]. More recently, the Vascular Impairment of Cognition Classification Consensus Study (VICCCS) guideline has been proposed [198]. Both working groups claim neuropsychological testing to demonstrate clinically significant deficits in at least one cognitive domain that result in impaired instrumental ADL. In addition, imaging evidence of cerebrovascular disease must accompany this demonstration. VaD is also frequently associated with psychological and behavioral symptoms (e.g., apathy, irritability, anxiety, sadness, disinhibition), with 81.1% showing at least one of them [172]. 

The neuropsychological assessment of patients with suspected vascular pathology should include core domains like executive function, attention, memory, language, and visuospatial function [198]. Research in this field suggests that predominant cognitive deficits in VCI are related to executive functions (i.e., verbal fluency, mental flexibility, processing speed, working memory, abstraction, reasoning), and attentional and slowed psychomotor function, with relative preservation of language and memory recognition tasks [145]. Although the cognitive profile of VaD is relatively established, controversy remains about the involvement of each cognitive domain. This may make sense in the context of the diversity of vascular etiologies and their severity, which makes it difficult to distinguish from other types of dementia [158,199]. The use of delayed recall tasks and evidence of predominant episodic memory impairment due to encoding and storage problems in patients with AD may help differentiate AD from VaD [199]. In this context, the use of semantic clustering indices in verbal learning tasks has demonstrated their ability to discriminate between AD and VaD [200,201]. In the study by Gaines et al. (2006), only the AD group showed a significant decrease in the ratio of semantic clustering from the last learning trial to delayed recall and poorer performance on other measures of semantic processing than controls (e.g., HVLT-R semantically related false positives, Boston Naming Test). On the contrary, executive dysfunction (i.e., planning, organizing, and monitoring behavior) and mental slowness should be prominent in VCI [158,159]. In terms of cognitive screening, the MoCA has shown good accuracy and reliability in detecting and differentiating mild VCI and VaD [202]. In addition, the Scientific Department of Cognitive Neurology and Aging of the Brazilian Academy of Neurology has proposed recommendations for the comprehensive cognitive, functional, and behavioral assessment of VaD [203]. Lastly, the Modified Hachinski Ischemic Score [204] may also assist the clinician in suspecting VaD and distinguishing it from AD.

### 4.5. Frontotemporal Dementia

Frontotemporal dementia (FTD) is a clinical–pathological condition comprising a heterogeneous group of clinical syndromes marked by progressive focal neurodegeneration of the frontal and anterior temporal lobes [205]. FTD includes three clinical syndromes based on its early and predominant symptoms: behavioral variant frontotemporal dementia (bvFTD), semantic dementia (SD), and progressive non-fluent aphasia (PNFA) [206]. FTD accounts for approximately 3–10% of all cases of dementia, with the behavioral variant (bvFTD) being the most common subtype [25]. An international consortium has developed revised guidelines for diagnosing this variant of FTD [148]. This working group defines the progressive deterioration of behavior and cognition as the core symptoms of bvFTD, which are represented by at least three of the following symptoms: (a) behavioral disinhibition; (b) apathy or inertia; (c) loss of sympathy or empathy; (d) perseverative, stereotyped, or compulsive/ritualistic behavior; (e) hyperorality and dietary changes; and (f) executive/generation deficits with relative sparing of memory and visuospatial functions.

Although these symptoms seem to outline a very clear clinical profile, the differential diagnosis between bvFTD and AD is complex. In this sense, a significant percentage of individuals with bvFTD show memory impairment (i.e., storage and consolidation), whereas a substantial percentage of those with AD show behavior changes and executive dysfunction, especially the behavioral/dysexecutive variant [140,169]. However, the disinhibition symptoms exhibited by patients with bvFTD in early and advanced stages can serve as a highly valuable measure to distinguish them from patients with AD [141].

It has also been observed that patients with bvFTD perform significantly worse than patients with AD on phonological (vs. semantic) fluency tests, but significantly better on tests of memory and visuospatial abilities [169], although this trend is not observed in all studies [141]. An analysis of performance characteristics (i.e., error types) could also enhance the distinction between FTD and AD [160,207]. Accordingly, these authors claim that patients with FTD typically exhibit features associated with frontal lobe dysfunction, such as concrete thinking, perseveration, confabulation, and poor organization, which may be associated with impaired performance on a variety of neuropsychological tests. Finally, patients with bvFTD show a more pronounced impairment in emotion processing than patients with AD, which includes failing to recognize facial expressions of basic emotions [169,208]. The screening tests and neuropsychological batteries that may assist the clinician in characterizing bvFTD are the INECO Frontal Screening (IFS) [209], the FRONTIER Executive Screen (FES) [210], the Executive and Social Cognition Battery (ESCB) [211], and the Frontal Assessment Battery (FAB) [212].

## 5. Discussion

This review reaffirms the usefulness of neuropsychological assessment in the early detection of cognitive changes associated with progression to dementia and the characterization of its different forms. However, this field continues to evolve, and neuropsychologists still face several challenges. For instance, the cognitive markers of preclinical stages of neurological diseases causing dementia are not yet operationalized, making it difficult to delineate clinical guidelines that streamline the detection of early signs of CI in healthcare settings [70]. Therefore, more efforts are being made to characterize cognitive decline from its earliest stages (i.e., SCD), delineating those profiles that are more susceptible to progression to MCI and AD dementia (e.g., [213]). In addition, research on these subtle early symptoms has focused primarily on AD, neglecting other cognitive trajectories that may be characteristic of other forms of dementia progression. It should be noted that SCD has been associated with psychological and personality factors (e.g., stress, neuroticism) [214], lifestyle and health habits [215,216,217], and cognitive reserve [218,219]. However, no conclusive causal relationships have been described, nor how these factors are associated with the SCD subtype [220]. 

Primary care is a critical setting for detecting the first cognitive concerns reported by patients and/or their families. These professionals face additional barriers to detect CI, such as lack of time or expertise in cognitive functioning and its assessment [25,27]. For this reason, there is a critical need to promote accessible training programs and develop sensitive, standardized, and easy-to-use dementia screening tests [129,221]. In addition, many traditional neuropsychological tests are unsuitable for diverse populations due to their reliance on school-based skills (e.g., reading and writing), the need for culturally dependent skills, and the lack of representative norms, which may favor the occurrence of false-positive cases of CI in low educated and/or non-Caucasian individuals [30,222,223,224]. Therefore, in addition to the training of primary care professionals, more efforts should be made to adapt the cognitive assessment practices for older adults, a population that is highly diverse in terms of culture, language, and education [80]. 

The early detection of dementia could lead to savings from delayed institutionalization, making it a potential cost-effective investment [72,225]. For example, it could provide early access to available pharmacological and psychosocial treatments. Nowadays, cholinesterase inhibitors such as donepezil, rivastigmine, and galantamine are used to manage mild to moderate symptoms, while glutamate antagonists such as memantine is prescribed for moderate to severe AD symptoms (see [226,227,228,229] for a review). Moreover, anti-amyloid immunotherapy in symptomatic AD (i.e., reducing the neurotoxic effects of Aβ) and other approaches (e.g., inhibiting microglial receptors, reducing inflammation) in the preclinical stage of AD have gained interest [53,230,231]. However, the relationship between Aβ and clinical progression remains unclear, and no protective or regenerative drug has yet been identified [231,232,233]. In this context, psychosocial interventions that emphasize patients’ lifestyle factors and behaviors (e.g., physical exercise, relaxation, art therapy, sensory and behavioral interventions) are receiving more attention [234,235]. In the absence of curative treatment, there is still an open debate about the need for widespread “early diagnosis” in the general population [71,78]. Instead, “timely diagnosis” is proposed, allowing people to be diagnosed at the time of symptom onset. Brooker et al. [78] argue that this would maximize the benefits and reduce the harm associated with earlier diagnosis, including reducing the stigma of dementia, recognizing how the diagnosis may affect subsequent psychosocial adjustment, and providing post-diagnosis support for individuals and their families. Given the current limitations for detecting CI on time, it is worth asking whether this debate makes sense [236,237,238]. To this end, collaborative and transdisciplinary working groups are needed to provide early professional support to patients and caregivers, including diagnostic assistance and holistic interventions [72,79,239].

In specialized contexts, a more comprehensive assessment is generally required, consisting of a neuropsychological toolset that focuses on different cognitive domains (e.g., language, memory, attention), ADL, and behavioral and emotional characteristics [49]. Researchers are coming closer to defining the cognitive profile characteristics of different types of dementia. However, cognitive signs often overlap between pathologies, making it difficult to discern the underlying etiology. The social, emotional, and behavioral symptoms (e.g., emotional lability, personality changes, abnormal social behavior), known to be common in neurodegenerative disorders, are usually addressed in a complementary manner [162,240]. Incorporating variables such as social cognition (in line with DSM-5 guidelines), emotional fluctuations, or disruptive behavior into standard neuropsychological assessment may provide relevant information to guide differential diagnosis and recommendations for daily functioning [221].

As technology has become more accessible, interest in adapting neuropsychological assessment to the digital format has grown rapidly in research settings [221]. For instance, digital devices could streamline standardization processes across larger populations or extend the monitorization of cognitive–functional changes beyond the clinician’s office. However, there are still numerous challenges that hinder the implementation of these new tools in clinical practice. These include a lack of consensus among experts, difficulties in interpreting results, the digital divide among older adults, ethical and privacy concerns, or tool updates that may affect the psychometric properties of the tests used [241,242]. While digital assessments, especially those that are supervised, demonstrate good concurrent validity and expected associations (i.e., with biomarkers, age, and clinical status), they may show insufficient comparability with traditional in-person versions, suggesting that the underlying constructs or difficulty levels are slightly different [243,244]. Ultimately, digital technology offers a promising approach for the early detection of clinical changes. Nonetheless, further consensus in terms of development and implementation are required according to the clinical and scientific principles underlying a standardized neuropsychological assessment [245].

This research is not without limitations. As a narrative review, the selection of the studies relies on the authors’ criteria, which partially limits the generalizability of the results. However, this research was carried out by experts in the field who tried to reflect the current state of the art based on extensive and recognized international databases. Although this narrative review lacks the methodological rigor of a systematic review, it may be a better option to address a specific topic in a broader way [33]. Moreover, the quality of the studies included in this review was not explicitly assessed. Nevertheless, we have followed the QUADAS and SANRA recommendations to ensure the appropriateness of the studies included in this review [32,33]. Therefore, we provide a useful comprehensive overview of the early cognitive changes associated with dementia for researchers and clinicians, including differential characteristics of dementia subtypes. In any manner, it is recommended to apply the information contained in this review according to the characteristics of the sociocultural context. 

## 6. Conclusions

Neuropsychological measures can provide reliable indicators of the presence of cognitive decline, while being cost-effective and minimally invasive. Clinical and experimental neuropsychological research is beginning to uncover the earliest preclinical cognitive changes that might predict the subsequent development of dementia. Moreover, it has delineated different cognitive profiles that distinguish AD from other age-associated neurodegenerative disorders, which enhances an accurate differential diagnosis of dementia subtypes. However, there are many challenges to overcome in the field. Study populations are becoming more diverse in terms of education, language, and culture, emphasizing the need to adapt the assessment strategies. The rapid advance of technology offers promising test application formats, although some barriers need be reconsidered if valid assessments are to be made. Finally, neuropsychological assessment plays a fundamental role in characterizing the early stages of cognitive decline, but there should be a commitment to developing harmonized procedures that facilitate the universal use of such assessment in different clinical contexts and research protocols.

## 7. Future Directions

Neuropsychological assessment plays a key role in detecting the early clinical signs of CI. However, prospective studies are still needed to investigate the preclinical cognitive profile underlying each of the neurodegenerative diseases that cause dementia in combination with biomarkers that accurately measure the progression of brain changes. To this end, expanding the study of cognitive performance at early stages of non-AD neurodegenerative diseases, such as VaD and FTD, is also necessary. Understanding the cognitive changes that precede dementia may enable neuropsychologists to develop and validate novel cognitive paradigms, which serve as frameworks for designing sensitive tasks to these early alterations. In this context, special attention should be paid to covariates that have been shown to influence cognitive performance (e.g., literacy, cultural background, lifestyle factors). In addition, each neuropsychological test should present normative data adapted to the target populations. Ultimately, characterizing the cognitive and psychological profiles of individuals at higher dementia risk (e.g., individuals with SCD) will facilitate the development of accurate tests to detect subtle CI at its earliest stages. Furthermore, these profiles may help to improve tailored prevention and intervention strategies.

Regarding dementia diagnosis, harmonizing protocols to different contexts (i.e., primary care, specialized medicine, memory clinics, or laboratories) could reduce the heterogeneity of methods across research studies and clinical approaches. These protocols should then consider the specificities of each application setting (e.g., application time, professionals’ training). Further, more studies are needed comparing the clinical utility of screening and diagnosis protocols in non-specialized (e.g., primary care) and specialized (e.g., memory clinics) settings. Regarding assessment protocols in specialized settings, it is important to emphasize the need to include emotional (e.g., apathy, lability), behavioral (e.g., disinhibition, agitation), and social (e.g., social cognition) variables that may be associated with incipient CI. Lastly, quantitative and qualitative assessment of cognitive domains should be accompanied by an analysis of emotional responses (e.g., frustration, catastrophizing, indifference). Attention to psychological and socio-behavioral symptoms, in addition to cognitive variables, can provide clinicians with highly relevant information to guide differential diagnosis and recommendations for daily functioning.

In-person neuropsychological assessment is considered the gold standard for the clinical diagnosis and characterization of individuals with CI, but digital evaluation and artificial intelligence (AI) may certainly play an important role in the future. Basically, digital assessment can potentially reduce the burden on the evaluator, allowing cost-effective data collection and improving standardization methods, which makes it promising for CI screening. Videoconferencing may be also interesting for vulnerable individuals or those living in remote areas (see [244] for a review). Similarly, AI can provide an opportunity to reach these populations by offering remote assessments through digital tools such as chatbots or avatars [246]. In addition, the use of AI techniques (e.g., machine learning algorithms) may assist clinicians to interpret neuropsychological data, making diagnostic decisions and predicting cognitive outcomes [247,248,249]. Ultimately, these novel tools for evaluating and analyzing information can improve personalized assessment and intervention strategies. To this end, it will be important to adopt scientifically and ethically sound practices in the development and adoption of digital health assessment strategies. The collaboration of experts from other disciplines, such as computer science, statistics, or even legal experts, will be crucial to ensure privacy and ethical issues.

## Figures and Tables

**Table 1:** A comparison of the different criteria for predementia constructs.

	Ebly et al., 1995 [17]	Petersen et al., 1999 [10]	Winblad et al., 2004 [12]	DSM-5 2013 [4]	ICD-11 2019 [18]
Designation	Cognitive impairment no dementia (CIND)	Mild cognitive impairment (MCI)	Mild cognitive impairment (MCI)	Mild neurocognitive disorder (mild NCD)	Mild neurocognitive disorder (mild NCD)
Individual’s subjective complaints	O	Memory loss	Cognitive Concerns	Cognitive Concerns	Cognitive Concerns
Informant complaints	O	O	O	O	O
Professional suspicion	O	-	-	O	O
Objective memory impairment	O	X	O	O	O
Non-amnestic impairment	O	-	O	O	O
Social cognition impairment ^a^	-	-	-	O	O
Functionality	Variable	Preserved	Independent, less efficient at complex activities	Staying independent requires strategies	Mild decline in complex activities
Rule out	Dementia, delirium, psychiatric conditions	Dementia	Dementia	Delusions, mental disorders	Dementia, delusions, mental disorders, substance use

Notes: O, optional; X, mandatory; -, not specified or not considered; ^a^ social cognition: cognitive processes involved in interacting with and understanding other people (e.g., emotion perception, theory of mind, social knowledge of rules and roles) [19].

**Table 2:** Cognitive screening instruments.

Application Time	Test	Main Components	Free Use	Validation Data ^a^		W. ext. ^b^	Refs.
				C	P		
<5 min	AMTS-Hodkinson	Short MS	Yes	Yes	No	++	[101]
	CSI-D	Short MS + Informant	Yes	Yes	Yes	+++	[102]
	CDT	EF, VS	Yes	Yes	Yes	+++	[103,104]
	Delayed-Recall	ME (logical/visual)	Yes	Yes	Yes	+++	[50,105]
	GPCOG	OR, WM + CDT + Informant	Yes	Yes	No	+	[106]
	Mini-Cog	ME + CDT	Yes	Yes	Yes	+++	[107]
	MIS	ME (verbal delayed + cued)	Yes	Yes	Yes	++	[108]
	Phototest	ME (visual)	Yes	Yes	No	+	[109]
	SPMSQ-Pfeiffer	Short MS	Yes	Yes	No	++	[110]
	Short IQCODE	ME + Informant	Yes	Yes	Yes	++	[111]
	Verbal fluency	EF, LA	Yes	Yes	Yes	+++	[50,112,113]
	6-CIT	OR, AT, WM, ME	Yes	Yes	Yes	++	[114]
5–10 min	MMSE	MS standard	No	Yes	Yes	+++	[90,92,93,95]
	MoCA *	OR, AT, LA, ME, EF, VS + CDT	No	Yes	Yes	+++	[115]
	QMCI	OR, AT, ME, EF + CDT	Yes	Yes	No	+	[116]
	TMT (A&B)	AT, EF	Yes	Yes	Yes	+++	[117,118]
	RUDAS	ME, PR, LA, JU, GN	Yes	Yes	No	+	[119,120]
	7 Min Screen	OR, ME, EF, VS	Yes	Yes	No	++	[121,122]
>15 min	ACE-R	AT, ME, VF, LA, VS	Yes	Yes	Yes	+++	[123]
	3MS	Long MS	Yes	Yes	Yes	+	[96]

Notes: ^a^ Validations performed in clinical (C) and/or population-based samples (P); ^b^ W. ext.: world extension and language use, from low (+) to great (+++: Chinese, English, Spanish, and other language), obtained from [124]. * May require more time. Test abbreviations: ACE-R: Addenbrooke’s Cognitive Examination Revised; AMTS: Abbreviated Mental Test; CDT: Clock Drawing Test. CSI-D: Community Screening Instrument for Dementia; GPCOG: General Practitioner Assessment of Cognition; Mini-Cog: Mini-Cognitive Assessment Instrument; MIS: Memory Impairment Screen; MMSE: Mini-Mental State Examination; MoCA: Montreal Cognitive Assessment; SPMSQ: Short Portable Mental Status Questionnaire; Short-IQCODE: Informant Questionnaire on Cognitive Decline in the Elderly; QMCI: Quick Mild Cognitive Impairment screen; RUDAS: Rowland Universal Dementia Assessment; 3MS: Modified Mini-Mental State; 6CIT: 6-item Cognitive Impairment Test. Domains: AT: attention; EF: executive functions; GN: gnosis; JU: judgement; LA: language; ME: memory; MS: mental status; OR: orientation; VF: verbal fluency; VS: visuospatial; WM: working memory.

**Table 3:** Summary of neuropsychological performance by dementia type.

	AD	PD-D	DLB	VaD	bvFTD
Memory	Decreased ability to learn new information and a marked deficit in long-term recall, even with cues [134,135].	Impaired free recall memory, which usually improves with cueing [136]. The verbal and non-verbal memory deficits are usually related to executive dysfunction [137].	May not be persistent at early stages, but becomes evident as the disease progresses [138]. Free recall is often impaired, but tends to improve with cues [136].	Difficulties in learning new information, probably related to attention or focus problems [139].	Amnesia may be present and appears to be a combination of both executive-mediated and storage-based memory impairments [140,141].
Attention	Decreased ability to concentrate and focus. Affected in early stages, especially in individuals with young onset and atypical syndromes [142].	Impaired and may fluctuate, more so than in AD [143].	Pronounced variations in attention and alertness [144]. Prominent deficits in selective, divided, and sustained attention [145,146].	Reduced choice reaction times and accuracy in sustained attention compared to AD patients [147].	Distractibility and attention deficits are often reported [148,149].
Language	Impaired language skills (e.g., anomia, comprehension difficulties) associated with semantic memory deficit [150,151].	Naming is often impaired to a variable degree, yet frank language impairments are not present [136].	Deficits in comprehension, word production, spontaneous speech, and reading, possibly secondary to semantic memory, visuoperceptual, and executive deficits [152].	Impairments in this area, while uncommon, can vary greatly depending on the type, extent, location, and severity of CVD [153].	Some may show impaired word or object knowledge, motor speech deficits, and grammatical deficits in language production or comprehension [148].
Executive functions	Deficits in working memory, abstraction, conceptual reasoning, planning, fluency, organization, and mental flexibility, which may lead to social alterations, difficulties in instrumental ADL, depression, and anosognosia [154,155].	Deficits in internal control of attention, mental flexibility, planning, inhibitory control, and decision-making tasks [156]. Most impaired and fastest decline compared to AD and DLB counterparts [157].	Significant deficits in initiation, perseveration, mental flexibility, and working memory [145,146]. Faster decline in comparison to AD counterparts [157].	Disturbance in frontal-executive functions (i.e., planning, organizing, monitoring behavior) is often the more salient feature [158]. Cognitive planning and mental flexibility are considerably affected even in the first stages [159].	Greater deficit in planning, mental flexibility, inhibition, and abstraction skills than in other cognitive abilities, which may lead to concrete thinking, perseveration, confabulation, and poor organization [160,161].
Visuospatial and visuoconstructive skills	Deficits in visual discrimination, analysis, spatial judgment, and perceptual organization, which can eventually lead to spatial disorientation in daily life [162,163]. Impairment in both graphomotor (drawing) and non-graphomotor (building and assembling) tasks [164].	Low performance in visual discrimination, object perception, and visuoconstructive abilities [165].	Salience of impaired visual processing [138,145,146]. Lower performance in object discrimination, overlapping figures, and visual counting tasks, and faster decline than in AD [157,166,167].	Significant disturbance of visuospatial abilities, including both object and spatial perception [168].	Relatively preserved in the early stages [169]. In advanced stages, there is a decline in visuospatial function that is less pronounced than in AD counterparts [170].
Neuropsychiatric symptoms	Personality changes, anxiety, depression, emotional lability, agitation, delusions [171].	Apathy, changes in personality and mood, hallucinations, delusions [143].	Visual hallucinations, depression, apathy, anxiety, delusions [144].	Apathy, irritability, anxiety, sadness, disinhibition [172].	Disinhibition, apathy, perseverative, stereotyped, compulsive behavior, hyperorality [148].

Notes: AD, Alzheimer’s disease; ADLs, activities of daily living; bvFTD, behavioral-variant frontotemporal dementia; CVD, cardiovascular disease; DLB, dementia with Lewy bodies; PD-D, Parkinson’s disease dementia; VaD, vascular disease.
